# Dynamic Imaging of Transferrin Receptor Molecules on Single Live Cell with Bridge Gaps-Enhanced Raman Tags

**DOI:** 10.3390/nano9101373

**Published:** 2019-09-25

**Authors:** Qinnan Zhang, Jiaosheng Li, Ping Tang, Xiaoxu Lu, Jindong Tian, Liyun Zhong

**Affiliations:** 1 Guangdong Provincial Key Laboratory of Nanophotonic Functional Materials and Devices, School of Information and Photoelectric Science and Engineering, South China Normal University, Guangzhou 510006, China; zhangqinnan520@126.com (Q.Z.); ljsheng1989@126.com (J.L.); tangping@m.scnu.edu.cn (P.T.); hsgdzlxx@scnu.edu.cn (X.L.); 2 College of Physics and Optoelectronic Engineering, Shenzhen University, Shenzhen 518060, China; jindt@szu.edu.cn

**Keywords:** surface-enhanced Raman scattering, bridge gaps-enhanced Raman tags, SERS-based imaging, nanoprobe

## Abstract

A metal nanoparticles-based surface-enhanced Raman scattering (SERS) technique has been developed for biosensing and bioimaging due to its advantages in ultra-narrow line width for multiplexing, ultra-high sensitivity and excellent photostability. However, the “hotspots” effect between nanoparticles usually leads to unstable and nonuniform Raman enhancement, and this will greatly reduce the quality of SERS imaging. In this study, we employ the bridge gaps-enhanced Raman tags (BGERTs) to perform SERS imaging, in which BGERTs can not only reduce the influence of the “hotspots” effect between nanoparticles on Raman signal intensity but provide a great Raman enhancement when the Gold (Au) shell is thick enough. Based on BGERTs and its conjugation with the thiol-terminated polyethylene glycol (PEG) and transferrin, we construct a targeted Transferrin (TF)-PEG-BGERTs SERS nanoprobe and achieve the dynamic imaging of transferrin receptor (TfR) molecules on a single live cell, in which the role of transferrin-conjugated PEG-BGERT is for targeting TfR molecules located in cellular membrane surface. Importantly, this BGERTs-based SERS imaging could potentially provide a useful tool for studying the precise mechanism during the receptor-mediated nanoparticles endocytosis or cell proliferation, apoptosis, and other complicated molecular events.

## 1. Introduction

Many important cellular events, such as cell proliferation, apoptosis, and other complicated molecular events, involve molecular behavior characterization and multimolecular interaction occurring on a nanoscale, and how to achieve dynamic imaging of cellular events in a single cell level is becoming increasingly required in cell and molecular biology sciences [1,2,3,4,5,6,7]. The transferrin receptor (TfR), is located in the cell membrane surface and plays an important role for transporting ferric ion and is an accessible portal for the cancer drug delivery due to the expression level of TfR on cancer cell being high, relative to the normal cell [8,9]. The dynamic imaging of TfR molecules in a single cell will provide reference data for the targeting therapeutic solution via the TfR system.

Owing to the advantages in ultra-narrow line width for multiplexing, ultra-high sensitivity and excellent photostability, Gold (Au) nanoparticles-based surface-enhanced Raman scattering (SERS) [10,11,12,13,14,15,16,17] is becoming a potential technique for long-time imaging of biomolecules in a single cell [18,19,20,21,22,23]. The imaging quality with SERS-active nanoparticles depends on the stability and uniformity of Raman enhancement, and linear correlation between SERS intensity and probe concentration. Though the near-field “hotspots” effect between SERS-active nanoparticles can provide strong Raman enhancement when the interval distance between nanoparticles is reduced, due to the micro-domain on plasma membrane or biomolecules aggregation, the near-field “hotspots” effect between nanoparticles will significantly affect the stability, uniformity, linear correlation between SERS intensity and probe concentration, and then lead to the unreliable imaging result [24,25].

In this study, the influence of the “hotspots” effect between SERS-active Au nanoparticles on Raman signal intensity is discussed, in which the bridge gaps-enhanced Raman tags (BGERTs) with hidden tags are employed for SERS imaging, thus, the stability of the Raman signal can be protected from the biochemical environment by modified medium or metal shell [26,27,28,29,30]. Specifically, it is found that BGERTs with 15 nm Au shell can not only effectively reduce the influence of the “hotspots” effect between nanoparticles on Raman signal intensity, but provide a great Raman enhancement, which is an important guarantee to achieve SERS imaging with high quality. In addition, we employ the parameter of signal-to-noise ratio (SNR) to evaluate the quality of SERS imaging. Specifically, based on BGERTs and its conjugation with the thiol-terminated polyethylene glycol (PEG) and transferrin, we construct a targeted Transferrin (TF)-PEG-BGERTs SERS nanoprobe and achieve the dynamic imaging of TfR molecules on a single live cell, in which the role of transferrin-conjugated PEG-BGERT is for targeting TfR molecules located in the cellular membrane surface and the nanoprobes internalization is implemented in a TfR-mediated way. Importantly, this BGERTs-based SERS imaging could potentially provide a useful tool for studying the precise mechanism during the receptor-mediated nanoparticles endocytosis or cell proliferation, apoptosis, and other complicated molecular events.

## 2. Materials and Methods

### 2.1. Synthesis of Functionalized Bridge Gaps-Enhanced Raman Tags (BGERTs) and Surface-Enhanced Raman Scattering (SERS) Imaging

All chemical reagents were obtained from commercial suppliers and used without further purifications. L-ascorbic acid (AA, >99.9%, Macklin Biochemical Co., Ltd., Shanghai, China), 1,4-benzenedithiol (BDT, >98%, Macklin Biochemical Co., Ltd., Shanghai, China), sodium borohydride (NaBH_4_, 99%) and cetyltrimethylammonium chloride (CTAC, 99.0%, Macklin Biochemical Co., Ltd., Shanghai, China). Hydrogen tetrachloroaurate trihydrate (HAuCl_4_·3H_2_O, 99.9%, Merck KGaA, Darmstadt, Germany).

**Figure 1:**
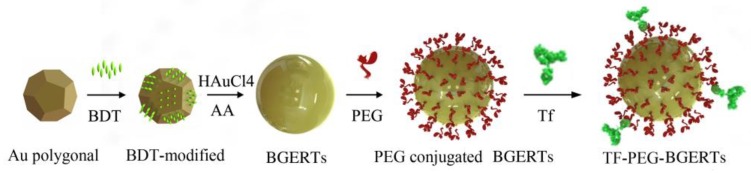
Sketch for preparing TF-PEG-BGERTs nanoprobe.

Figure 1 showed the sketch for preparing TF-PEG-BGERTs nanoprobe. First of all, we prepared 20 nm Au polygonal cores, in which a seed solution was achieved according to the seed-mediated protocol [10,11,31], 10 mL of aqueous CTAC (0.2 M), 4.75 mL of water, and 250 μL of HAuCl_4_ (10 mM) with 600 μL of a NaBH_4_ solution (0.01 M) were mixed in a 50 mL Erlenmeyer flask under vigorous stirring. Second, 15 μL of the seed solution was added to the growth solution of 10 mL of CTAC solution (0.1 M) 300 μL of HAuCl_4_ (10 mM) and 60μL of ascorbic acid (0.1 M) under sonication. Third, the mixture was placed for 30 min, and the final concentration of Au was about 59 μg/mL.

Following this, we prepared the BGERTs referring to the Khlebtsov method [16]. First, 60 μL of BDT (2 mM) ethanol solution were respectively added into 2 mL of the above-prepared Au polygonal cores under sonication for 30 min. The BDT-modified cores were washed three times by centrifugation at 10,000 rpm for 10 min to remove the unreacted reagent, and then dispersed in 1 mL of aqueous CTAC (0.1 M). Second, 150 μL of BDT-modified polygonal cores solution were respectively added to the growth solution of 2 mL of CTAC (0.1 M), 100 μL of ascorbic acid (0.04 M), and 40 μL of HAuCl_4_ (10 mM) for 40 min. Finally, the BGERTs were placed in the darkroom at room temperature. The final concentration of Au was about 20 μg/mL. The excitation spectrum was measured with a UV-Visible spectrophotometer (Agilent 8453, Agilent, Santa Clara, CA, US). A transmission electron microscope (TEM, JEM2100, JEOL, Tokyo, Japan) was employed to present the nanostructure.

For preparing TF-PEG-BGERTs nanoprobe, 1 mL of BGERTs was washed three times by centrifugation at 4000 rpm for 10 min, and dispersed in 10 mL distilled water to remove CTAC. Then, 5 mL BGERTs solution was mixed with 1 mg of succinimidyl-polyethyleneglycol-thiols (NHS-PEG-SH, YongBio, Beijing, China) for 6 h to achieve the conjugation of PEG and BGERTs (PEG-BGERTs). Subsequently, the human transferrin (Jackson Immuno Research, West Grove, PA, USA) was employed to conjugate with the transferrin receptor (TfR) on HeLa cellular membrane surface. Finally, 1 mg of transferrin was added to PEG-BGERTs for 12 h, and the TF-PEG-BGERTs nanoprobe can be achieved.

For dynamic SERS imaging during TfR-mediated TF-PEG-BGERTs endocytosis in a single HeLa cell, the sample was prepared as following: First, 100 uL HeLa cells with the density of 1 × 10^5^/mL were cultured on the coverslip in a six-hole petri dish with 2 mL dulbecco’s modified eagle medium (DMEM) containing 10% fetal bovine serum (Hyclone, Jackson Immuno Research, West Grove, PA, USA), and 1% of the antibiotic Penicillin G (100 units/mL)/Streptomycin (100 μg/mL) (Gibco, Jackson Immuno Research, West Grove, PA, US) in the incubator with the temperature of 37 °C and CO_2_ of 5% concentration for 24 h. Then, the cell was grown and imaged on the coverslip as the substrate. Second, 300 μL PEG-BGERTs were conjugated with the transferrin named as TF-PEG-BGERTs, and 300 μL PEG-BGERTs without transferrin named as PEG-BGERTs was used as the control group. Third, the above two samples were respectively incubated with HeLa cells for 30 min and washed with phosphate-buffered saline (PBS) to remove the unreacted nanoprobe. After that, 2 ml DMEM was added in the culture dish. Finally, a Raman spectrometer (InVia Plus, Renishaw, Pliezhausen, Germany) attached to a Leica upright microscope equipped with a 50× objective, a 633 nm laser with the power of 0.17 mW and a charge-coupled device (CCD) detector with a spectral resolution of 0.99 cm^−1^ were employed to collect Raman spectrum of the measured sample, in which the Raman spectrum was implemented in the scan range of 200−1800 cm^−1^. In our study, the Raman spectral processing software was provided by the WiRE 4.3. apply innovation^TM^ (Renishaw plc, Pliezhausen, Germany).

### 2.2. Analysis of the Influence of the “Hotspots” Effect on the SERS Signal

Previous studies have demonstrated that near-field hotspots between metal nanoparticles provide strong Raman enhancement, and when the interval distance of nanoparticles is decreased, the intensity of the Raman signal will be sharply increased [24,25]. However, due to the “hotspots” effect between nanoparticles usually leading to the unstable and nonuniform Raman enhancement and the low linear correlation between probe concentration and SERS intensity, it is very difficult to achieve high-quality SERS imaging. 

To illustrate the influence of hotspots on the SERS signal, we employed the finite-different time-domain (FDTD) solution to simulate the electromagnetic (EM) field distribution of BGERTs pairs and Au nanospheres pairs, in which Yee cell size was set as 0.5 nm × 0.5 nm × 0.5 nm and the wavelength of excitation laser was 633 nm. The Au core size of BGERTs was set as 30 nm and the gap was set as 0.7 nm. In order to further discuss the influence of shell thickness on the “hotspots” effect, the Au shell thickness was set as 5 nm and 15 nm and the Au nanospheres size was set as 60 nm. Here, the permittivity was determined by the experimental value of the different frequency from the sample data mode of FDTD solution, and Au material data of the Chemical Rubber Co. (CRC) Handbook of Chemistry and Physics was employed for simulation. The EM distributions of nanoprobes with different interval distances were simulated, and the variation of EM enhancement can be used to analyze the influence of the “hotspots” effect on the tags SERS’ intensity.

Due to the Brownian movement of nanoparticles, they will collide randomly with each other and produce the “hotspots” effect. With the concentration of nanoparticles increasing, the collision frequency of nanoparticles will be increased, and the influence of the “hotspots” effect on the Raman signal intensity will become obvious. This will lead to the Raman signal intensity being not proportional to the probe concentration. Therefore, the consistency of the SERS signal intensity and probes concentration can be used for analyzing the influence of the “hotspots” effect on the quality of SERS imaging. To verify the simulation results, an experiment was performed: BDT-modified Au nanospheres with a size of 60 nm and BGERTs with a 15 nm Au shell were prepared, and the intensity variation of the SERS signal with the concentration of Au nanospheres or BGERTs were measured, respectively. The intensity of the Raman peak was defined as the peak area integral, and 2 mL Au nanospheres and BGERTs with the same concentration of Au at about 20 μg/mL were concentrated to 500, 250, 100 and 25 μL, respectively. A Renishaw InVia Raman spectrometer (InVia Plus, Renishaw, Pliezhausen, Germany) was employed to collect the Raman spectrum of the measured sample, in which the Raman spectrum was implemented in the scan range of 200–1800 cm^−1^. Raman spectral processing was performed with the software of Renishaw plc WiRE 4.3. apply innovation^TM^ (Renishaw plc, Pliezhausen, Germany), in which the fluorescence background of the Raman spectrum was estimated by using the fifth order polynomial fitting and then subtracted from the original spectrum.

### 2.3. Toxicity Testing

To assess the toxicity of BGERTs, first, for each well of 96-well plates, 100 uL HeLa cells with the density of 1 × 10^5^ cells/mL were cultured in dulbecco’s modified eagle medium (DMEM) containing 10% fetal bovine serum (Hyclone, Jackson Immuno Research, West Grove, PA, USA), and 1% of the antibiotics Penicillin G (100 units/mL)/Streptomycin (100 μg/mL) (Gibco, Jackson Immuno Research, West Grove, PA, USA) in the incubator with the temperature of 37 °C and CO_2_ of 5% concentration. Second, for comparison, three group samples were prepared as follows: (i) 10 uL bare BGERTs with the concentration of 0.01, 0.05, 0.1 mg/mL were respectively added to each well for 24 h and 48 h, (ii) 10 uL PEG-BGERTs with the concentration of 0.01, 0.05 and 0.1 mg/mL were respectively added to each well for 24 h and 48 h, and (iii) HeLa cells were cultured without any treatment as the control group. Finally, 10 uL cell count kit-8 (cck-8) solution were added to each well for 2 h, and the samples were measured by a microplate reader (iMark, Bio-Rad, Her- cules, CA, USA). The survival rate was defined as a ratio of *I*/*I*_control_, in which *I* denoted the absorption intensity at 450 nm wavelength of Hela cells co-cultured with BGERTs or PEG-BGERTs, and *I*_control_ was the absorption intensity at the 450 nm wavelength of the control group.

## 3. Results and discussion

### 3.1. BGERTs Characterization and SERS Imaging

**Figure 2:**
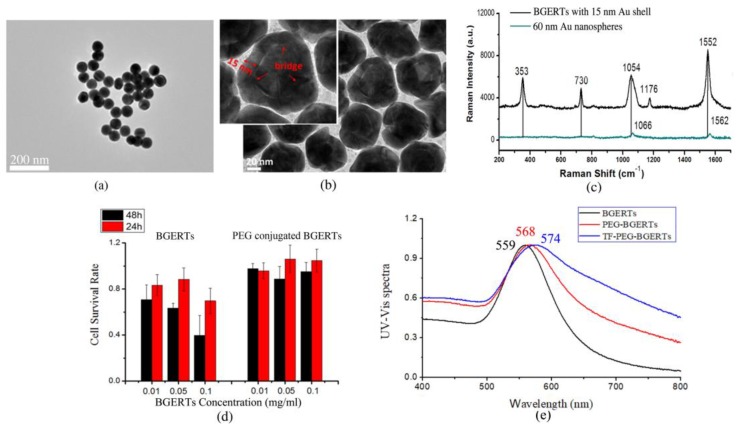
Transmission electron microscopy (TEM) images of (**a**) 60 nm Au (gold) nanospheres, (**b**) BGERTs with 15 nm Au shell, (**c**) Raman spectra of (**a**) and (**b**), (**d**) the survival rate of HeLa cells, in which HeLa cells were respectively cultured with the bare BGERTs and PEG-BGERTs with different concentrations, (**e**) the UV-Vis spectra of BGERTs, PEG-BGERTs and TF-PEG-BGERTs, respectively.

Subsequently, we prepared Au nanospheres with the size of 60 nm and BGERTs with a 15 nm Au shell. Figure 2a,b show the transmission electron microscopy (TEM) image of the prepared nanoparticles. It was observed that the size of Au nanospheres was about 60 nm and the size of the Au cores of BGERTs was about 30 nm, respectively. The corresponding Raman spectra are shown in Figure 2c, we can see that three strong characteristic peaks of BDT at 1054 cm^−1^ attributed to the phenyl-ring breathing mode (CH in-plane bending and CS stretching) [32], 1552 cm^−1^ attributed to the phenyl-ring stretching motion (8a vibrational mode) [32] and 353 cm^−1^, appeared in the Raman spectrum of BGERTs, meanwhile, two weak characteristic peaks at 1176 cm^−1^ attributed to the CH bending motion (9a vibrational mode) [32] and 730 cm^−1^ also were observed. Note that two characteristic peaks at 1054 and 1552 cm^−1^ appeared in the red shift, and a great broadening of the characteristic peak at 1054 cm^−1^ was observed. Raman signal intensity of the 60 nm Au nanospheres had only two strong characteristic peaks of BDT at 1066 cm^−1^ and 1562 cm^−1^ and a slight red shift, while the characteristic peaks at 1177 cm^−1^, 353 cm^−1^ and 730 cm^−1^ cannot be observed. These results demonstrated that the Raman enhancement of BGERTs was strong relative to Au nanospheres. To further verify whether that PEG and transferrin were successfully conjugated with BGERTs, we also measured the UV-Vis spectrum by a UV-Vis Spectrophotometer (Japan, Shimadzu, UV-2700), as shown in Figure 2e. We can see the absorption peaks of BGERTs, PEG-BGERTs and TF-PEG-BGERTs samples respectively appeared at 559 nm, 568 nm and 574 nm, the corresponding red shifts fully exhibited that the PEG and transferrin were successfully conjugated with BGERTs, respectively.

To facilitate the BGERTs-based SERS application in live cell research, we performed the corresponding cytotoxicity testing. First, BGERTs was wrapped by the NHs-PEG-SH with good biocompatibility, in which the sulfhydryl (-SH) was conjugated with BGERTs, and the BGERTs-modified PEG (NHs-PEG) was employed to conjugate with the specific molecules. Second, the cytotoxicity of BGERTs was evaluated by using the survival rate of the cell, as shown in Figure 2d. It was found that the survival rate of HeLa cells treated with bare BGERTs was lower than 90%, and along with the increasing of concentration or treatment time, the corresponding survival rate was further reduced. In contrast, the survival rate of HeLa cells treated with PEG-BGERTs was more than 90%. These results illustrated that the cytotoxicity of PEG-BGERTs on live cells can be ignored, further indicating that PEG-BGERTs were suitable for SERS imaging of live cells.

**Figure 3:**
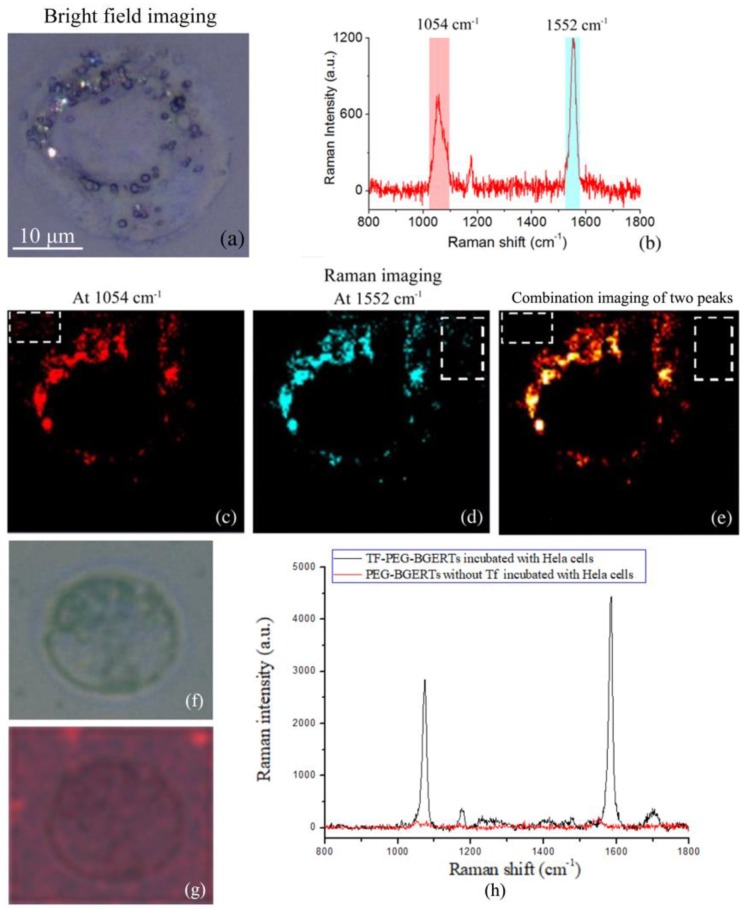
(**a**)Bright field imaging of a single HeLa cell, (**b**) Raman spectrum of BGERTs with two characteristic peaks at 1054 and 1552 cm^−1^, BGERTs-based surface-enhanced Raman scattering (SERS) imaging of the characteristic peaks at (**c**) 1054 cm^−1^, (**d**) 1552 cm^−1^, and (**e**) 1054 and 1552 cm^−1^, in which the white squares represented that the quality of SERS imaging of two characteristic peaks was greatly improved relative to the single peak due to the spurious signals and noises being reduced, (**f**) the bright field imaging of the control sample, (**g**) SERS imaging of the control sample of the characteristic peaks at 1054 and 1552 cm^−1^, (**h**) Raman spectra of the control group and the experimental group.

Further, using TF-PEG-BGERTs nanoprobes, we performed SERS imaging of TfR molecules on a single HeLa cell, as shown in Figure 3, in which the bright field imaging of a single HeLa cell was also given (Figure 3a), and the characteristic peaks at 1054 and 1552cm^−1^ were respectively chosen to achieve SERS imaging (Figure 3b–d), and their combination imaging of two characteristic peaks was employed to reveal the concentration variation of TF-PEG-BGERTs nanoprobes (Figure 3e). Interestingly, we can see that the quality of the combination imaging of two characteristic peaks was greatly improved relative to the single peak imaging due to the spurious signals and noises being reduced, indicating that BGERTs-based SERS imaging was suitable for biomolecule characterization with low concentration.

In addition, to further verify whether that the transferrin was successfully conjugated to PEG-BGERTs, an experimental result of the control group is shown in Figure 3f–h. We can see that two characteristic peaks at 1054 and 1552 cm^−1^ appeared in the TF-PEG-BGERTs, while no peak was observed in PEG-BGERTs. Further, the SERS imaging result of the peaks at 1054, 1552 cm^−1^ and their combination imaging, further demonstrated that the individual PEG-BGERTs without transferrin were difficult to be conjugated to the cell surface or internalized into the cell.

**Figure 4:**
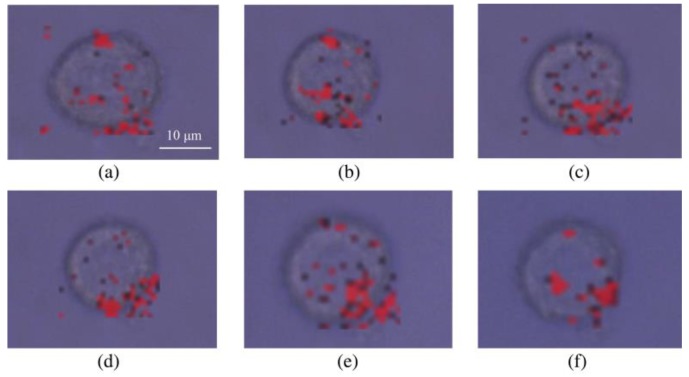
Dynamic imaging of transferrin receptor (TfR) molecules on a single HeLa cell at (**a**) 0 min, (**b**) 10 min, (**c**) 20 min, (**d**) 30 min, (**e**) 40 min, and (**f**) 50 min.

Here, the dynamic imaging of TfR molecules on a single HeLa cell is shown in Figure 4, in which the exposure time of each pixel was 10 ms. Clearly, the achieved SERS imaging result fully revealed excellent photostability and low cell damaging of TF-PEG-BGERTs nanoprobes. In addition, we can see that some TfR molecules were assembled on the cell surface, reflecting that the TfR-mediated nanoparticles endocytosis might be implemented by the aggregation of TfR molecules. In a word, the distribution and aggregation information of TF-PEG-BGERTs in a HeLa cell could potentially provide important reference data for studying the precise mechanism of TfR-mediated nanoparticles endocytosis. However, the micro-domain on the plasma membrane or the aggregation of biomolecules would cause the aggregation of nanoprobes, the “hotspots” effect between nanoparticles would restrict the realization of high-quality SERS imaging due to its influence on the linear correlation between SERS intensity and probe concentration. The imaging speed would also affect the imaging quality of SERS imaging. To solve these problems, we will discuss the influences of the “hotspots” effect on the tags SERS intensity and SERS-based imaging speed and quality in the following sections.

### 3.2. Analysis of the Influence of the “Hotspots” Effect on the Tags’ SERS Intensity

Due to the micro-domain on the plasma membrane or biomolecules aggregation, the near-field “hotspots” effect between SERS-active nanoparticles will provide strong Raman enhancement when the interval distance of nanoparticles is reduced. Following this, we performed the simulation about the EM distributions of two BGERTs with different interval distances, and the intensity variation of EM enhancement was used to analyze the influence of the “hotspots” effect on the tags’ SERS intensity.

**Figure 5:**
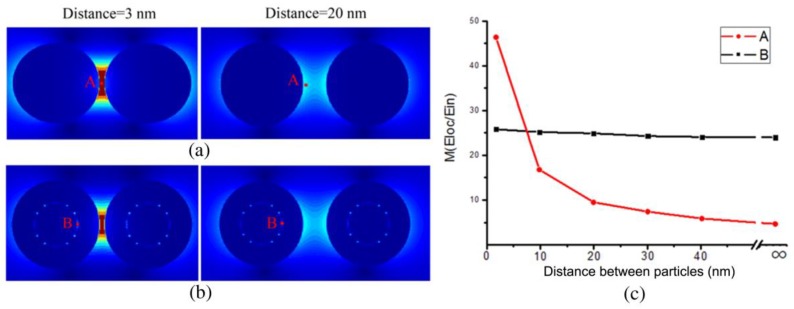
The simulated electromagnetic (EM) distributions of (**a**) two BGERTs with the interval distances of 3 nm and 20 nm respectively, (**b**) two Au nanospheres with the interval distances of 3 nm and 20 nm respectively, (**c**) the intensity variations of EM enhancement with the interval distances, in which A and B presented the reporter positions of Au nanospheres (**a**) and BGERTs (**b**), respectively.

First, we presented the simulated EM distribution of two BGERTs probes with the interval distances of 3 nm to 100 nm (Figure 5), respectively. We can see that the position of the reporter in Au nanospheres has great EM enhancement, and along with the interval distance increasing of two Au nanospheres, the corresponding EM enhancement factor was changed from 46.2 to 4.3 (Figure 5a,c). In contrast, the position of the reporter in BGERTs revealed the strong and stable EM enhancement factor, and along with the interval distance increasing of two BGERTs, the corresponding EM enhancement factor was changed only from 24 to 26 (Figure 5b,c), indicating the good stability of BGERTs-based EM enhancement.

**Figure 6:**
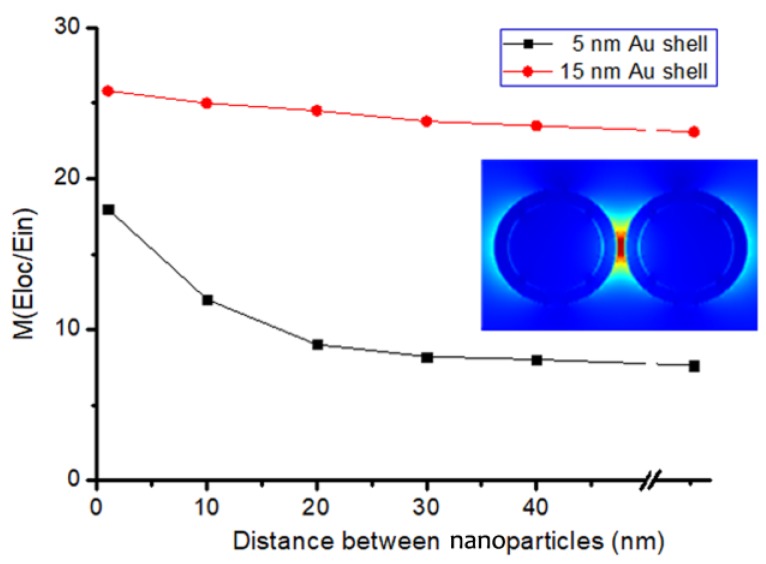
The influence of Au shell thickness of BGERTs on the SERS intensity.

In order to further analyze the influence of shell thickness on the "hotspot" effect, the EM field distributions of BGERTs with different Au shell thickness were simulated, in which the size of the core was set as 30 nm, and the Au shell thickness were set as 5 nm and 15 nm, respectively. The local electric field distribution of the above two BGERTs with the interval distance of 3 nm to 100 nm were calculated and the simulation results are shown in Figure 6. We can see that along with the increasing of the gold shell thickness, the Au shell can greatly reduce the influence of the “hotspots” effect between nanoparticles on Raman signal intensity. Meanwhile, a strong Raman enhancement was achieved. Clearly, this is a very important guarantee to achieve SERS imaging with high SNR.

**Figure 7:**
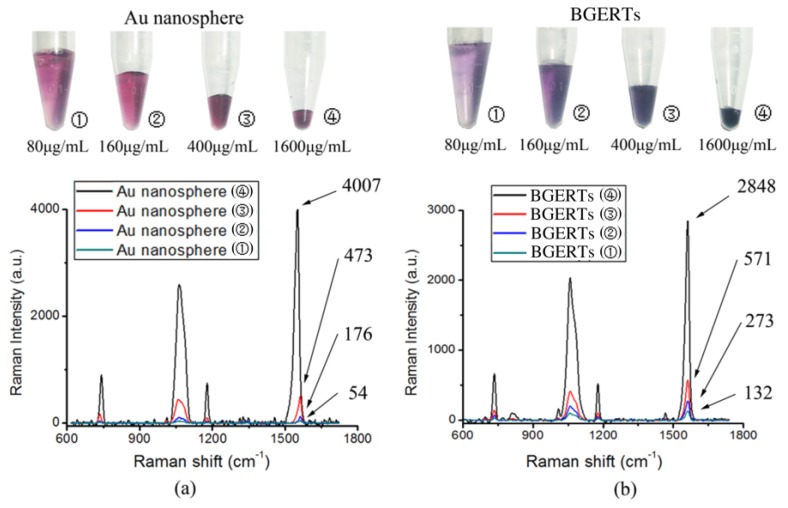
The influence of the nanomaterial concentration on the SERS signal intensity (**a**) Au nanospheres, (**b**) BGERTs.

Subsequently, Figure 7a, b shows the intensity variation of the SERS signal with the concentration of Au nanospheres and BGERTs respectively, in which the intensity of the Raman peak was defined as the peak area integral, and 2 mL Au nanospheres and BGERTs with the same concentration of Au about 20 μg/mL were concentrated to 500, 250, 100 and 25 μL, respectively. We can see in the case that the concentration of Au nanospheres were respectively 80, 160, 400 and 1600 μg/mL, the corresponding intensity ratio of the Raman peak at 1552 cm^−1^ was 1:5:20:103, indicating that the signal intensity is not directly proportional to the concentration of Au nanospheres, and in the case that the concentration of BGERTs were respectively 80, 160, 400 and 1600 μg/mL, the corresponding intensity ratio of the Raman peak at 1552 cm^−1^ was 1:2:4.9:20.5, exhibiting that the signal intensity was nearly proportional to the concentration of BGERTs. Clearly, this result further demonstrated that near-field hotspots between BGERTs have almost no influence on their SERS signal intensity, and this will supply the guarantee to achieve high-quality SERS imaging.

### 3.3. Analysis of BGERTs-Based SERS Imaging Quality

The quality of SERS imaging was closely related with the exposure time of Raman signal acquisition. In this study, in order to avoid cell damaging, the Raman spectrum acquisition of BGERTs was implemented at a low laser power of 0.17 mW, and the SNR was employed to evaluate the quality of SERS imaging, in which the SNR can be expressed as:

$$\mathrm{SNR}\;=10\times \log_{10}\frac{\mathrm{signal}\;\mathrm{power}}{\mathrm{noise}\;\mathrm{power}},$$

**Figure 8:**
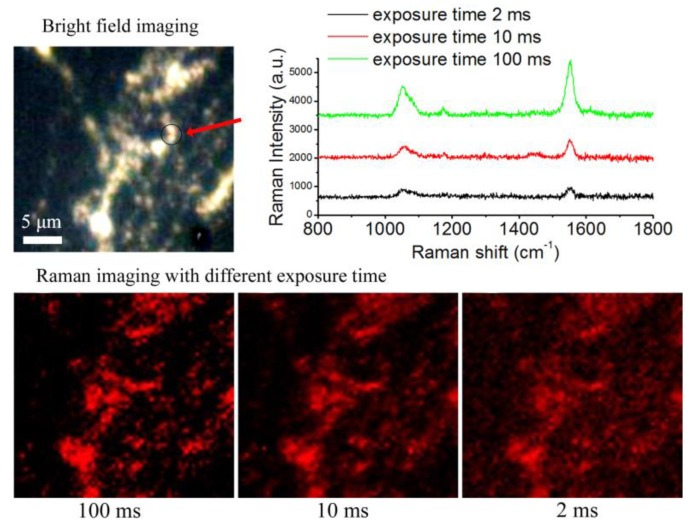
The SERS imaging result of BGERTs in different exposure times.

**Figure 9:**
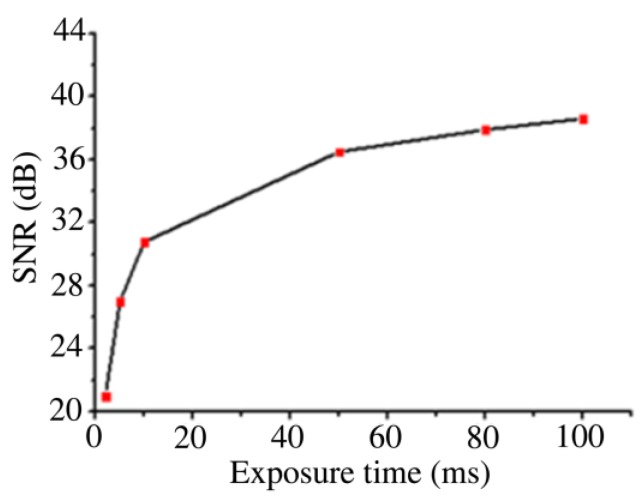
The signal-to-noise ratio (SNR) variation of BGERTs-based SERS imaging in different exposure times.

Figure 8 shows the SERS imaging results of BGERTs in different exposure times of 2, 10 and 100 ms, in which the characteristic peak at 1552 cm^−1^ was employed to perform SERS imaging, and the signal power was defined as the intensity square sum of each pixel at 1552 cm^−1^, the noise power was defined as intensity square sum of each pixel at 900 cm^−1^. Figure 9 presents the SNR variation of BGERTs-based SERS imaging with the exposure time. We can see that even in the case of low laser power (0.17 mW) and low exposure time (10 ms), the SNR of SERS imaging was still very high. This result demonstrates that the BGERTs-based SERS imaging was very suitable for long-term monitoring of dynamic processes.

## 4. Conclusions

In this study, we achieved the dynamic imaging of TfR molecules on a single live cell, in which the bridges and nanogaps between the Au core and Au shell reveal strong, stable and uniform Raman enhancement. Meanwhile, the near-field hotspots between the nanoparticles have almost no influence on the corresponding SERS signal intensity, and this will provide a guarantee for high-quality SERS imaging in a single live cell. Moreover, we constructed the targeted TF-PEG-BGERTs nanoprobe, in which the BGERTs were conjugated with the thiol-terminated PEG and targeted with the transferrin, and then the TF-PEG-BGERTs nanoprobe was internalized into the cell in a TfR-mediated way. Further, we employed the SNR to evaluate the quality of the BGERTs-based SERS imaging. The achieved results demonstrate that even if the illumination laser power and the exposure time are low enough, BGERTs-based SERS imaging still maintains a high quality, indicating that the BGERTs-based SERS nanoprobe was suitable for long-time monitoring of dynamic processes. Importantly, this BGERTs-based SERS imaging could potentially provide a useful tool for studying the precise mechanism during the receptor-mediated nanoparticles endocytosis or cell proliferation, apoptosis, and other complicated molecular events.
